# Loss of CXCR5 expression and monocyte epithelial–mesenchymal transition are blood‐borne signatures of sterile granulomatous diseases

**DOI:** 10.1002/cti2.70039

**Published:** 2025-06-03

**Authors:** Yuwei Hao, Anthea Anantharajah, Jane M Wells, Lyndell L Lim, Anthony JH Hall, Gary YJ Chew, Matthew C Cook

**Affiliations:** ^1^ Division of Immunology and Infectious Diseases, John Curtin School of Medical Research Australian National University Canberra ACT Australia; ^2^ Department of Immunology The Canberra Hospital Canberra ACT Australia; ^3^ Cambridge Institute for Therapeutic Immunology and Infectious Diseases University of Cambridge Cambridge UK; ^4^ Department of Immunology Wellington Hospital Wellington New Zealand; ^5^ Department of Ophthalmology The Canberra Hospital Canberra ACT Australia; ^6^ Royal Victorian Eye and Ear Hospital Melbourne VIC Australia; ^7^ Centre for Eye Research Australia The University of Melbourne Melbourne VIC Australia; ^8^ Department of Ophthalmology Alfred Hospital Melbourne VIC Australia; ^9^ Australian and New Zealand Intensive Care Research Centre, School of Public Health and Preventive Medicine Monash University Melbourne VIC Australia

**Keywords:** CXCR5, granuloma, sarcoidosis, tattoo, uveitis

## Abstract

**Objectives:**

Sarcoidosis is the exemplar sterile granulomatous disease and can affect any organ system. Tattoo uveitis (TU) resembles sarcoidosis clinically and histologically but is distinguished by the absence of systemic lymphadenopathy, with inflammation restricted to skin and eyes. In this study, our objectives were, first, to resolve whether TU is a subset of sarcoidosis or a different antigen‐driven condition and, second, by comparing TU and sarcoidosis, to identify blood‐borne signatures of active and quiescent sterile granulomatous diseases.

**Methods:**

We recruited patients with active and inactive TU, sarcoidosis and healthy controls on whom we performed blood cell phenotyping and transcriptomics.

**Results:**

Unlike sarcoidosis, active TU is characterised by marked CXCR5 down‐regulation on B cells and CD4^+^ T cells that normalises on remission. TCR‐VDJ sequencing reveals an antigen‐driven response in sarcoidosis, but not in TU, with clonally expanded cytotoxic and terminally differentiated CD8^+^ effectors. Both active TU and sarcoidosis exhibit gene signatures of epithelial‐to‐mesenchymal transition (EMT) in circulating monocytes, whereas epithelioid macrophages are a hallmark of active granulomas.

**Conclusion:**

We have identified both shared and specific phenotypes in TU and sarcoidosis. Marked CXCR5 down‐regulation occurs in active TU and could explain the unique absence of lymphadenopathy. Both TU and sarcoidosis are characterised by inflammatory monocyte phenotypes and transcriptional signatures of EMT.

## Graphical abstract

Tattoo uveitis (TU) resembles sarcoidosis but is distinguished by the absence of systemic lymphadenopathy despite inflammation of skin and eyes. In this study, we compared blood‐borne signatures of these two diseases and found that both are characterised by inflammatory monocytes and transcriptional signatures of epithelial–mesenchymal transition, but CXCR5 down‐regulation occurs in TU, while sarcoidosis appears to be antigen‐driven.

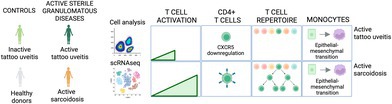

## Introduction

Granulomas typically arise in response to antigens that cannot be eradicated after phagocytosis, either because of physical characteristics of the antigen, a defective host immune response or as an adaptation to deal with intracellular pathogens.^1^, ^2^ In addition, sterile granulomas arise in a number of primary inflammatory disorders. Sarcoidosis is the most prevalent of these but others include tubulointerstitial nephritis and uveitis (TINU), Blau syndrome and Crohn's disease.^3^, ^4^, ^5^, ^6^

With only about 30 reported cases, Tattoo uveitis (TU, also termed Tattoo‐associated granulomas with uveitis) is a rare granulomatous disease that presents as localised inflammation confined to tattoos and the uveal tract.^5^ It remains unclear whether TU represents a limited form of sarcoidosis or a distinct antigen‐driven granulomatous disease. Both tattoo inflammation and uveitis can also occur in sarcoidosis, but almost any other end‐organ involvement can occur as well. By contrast, the clinical phenotype of TU appears homogeneous and stereotypic, with a close temporal association between tattoo acquisition and the onset of ocular and tattoo inflammation.^4^, ^5^ TU patients can be distinguished from sarcoidosis and other infectious granulomatous diseases by a striking absence of systemic lymphadenopathy^4^, ^5^ but no cellular biomarkers exist to distinguish TU from sarcoidosis.

Infective and non‐infective granulomas are characterised by peripheral T cells surrounding macrophages that undergo transformation to epithelioid cells, with formation of complex E‐cadherin‐containing adherens junctions as well as classical desmosomes.^1^, ^2^, ^7^, ^8^ Epithelioid granulomas can be driven by cytokines associated with Th2 response (IL‐4 and IL‐13), and enhanced by IL‐10 and TGF‐β.^1^, ^9^ Transcriptome analysis confirmed upregulation of genes essential for apical junctions and polarity, a process that resembles mesenchymal‐epithelial transition (MET).^7^

While single‐cell analysis has clarified the molecular events associated with macrophage epithelialisation during tissue granuloma formation, studies of sarcoidosis and other granulomatous conditions are often static observations and restricted to a snapshot in time and space of established granulomas.^10^, ^11^ In contrast, sterile granulomatous diseases are dynamic structures that fluctuate with disease activity and inflammatory complications in non‐lymphoid parenchyma. Pathology in granulomatous disease results from tissue derangement of lymph nodes and non‐lymphoid parenchyma by granulomas themselves. Pathology can also arise at sites distal to the primary granulomas, implying cell migration and granuloma dynamics are influenced by circulating immune cells. In a mouse model, deletion of circulating monocytes, via deletion or blockade of CCR2, led to regression of pre‐existing granulomas.^12^, ^13^ Based on this, we postulated that analysis of PBMCs from patients with either sarcoidosis or TU would complement granuloma tissue studies, clarify the relation between the two conditions and provide insights into the mechanisms underlying fluctuations between disease activity and quiescence. This has clinical significance, as PBMCs are easily accessible and can provide valuable biomarkers of disease activity and the mechanisms of distant complications.

Here, we report that the analysis of PBMCs from patients with active and inactive TU, sarcoidosis and healthy controls. We found a prominent TNF‐based inflammatory phenotype that paralleled disease activity in both TU and sarcoidosis. Remarkably, active TU was distinguished by a selective reduction in CXCR5 expression, which normalised with effective treatment. TCR sequencing further uncovered an antigen‐driven response in sarcoidosis, characterised by clonally expanded CD8^+^ cytotoxic effectors, a feature absent in TU. In contrast to reported signatures from epithelioid macrophages in granulomas, we observed epithelial–mesenchymal transition in blood monocytes.

## Results

### Active tattoo uveitis is associated with CXCR5 down‐regulation and undetectable cTfh cells

**Figure 1:**
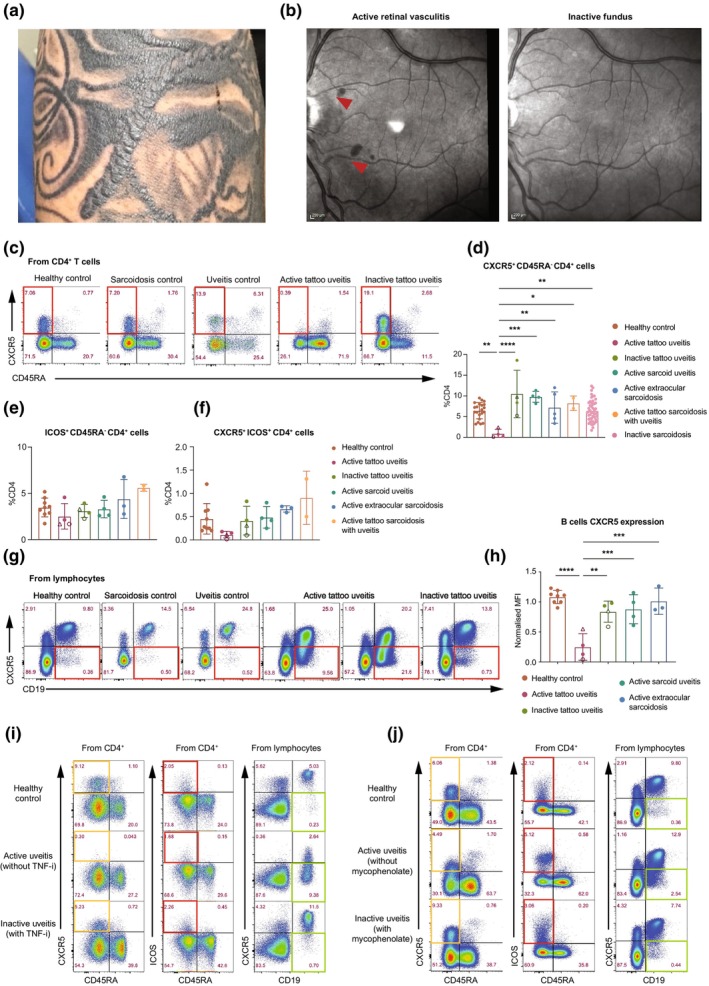
CXCR5 down‐regulation is associated with active inflammation in tattoo uveitis. **(a)** Tattoo elevation and induration in multiple black pigmented tattoos in a male patient; symptoms of ocular inflammation (bilateral anterior uveitis) developed within weeks of tattoo inflammation. **(b)** Optical coherence tomography (*en face* view) demonstrating retinal infiltrates (arrowheads) during active ocular disease in the patient which resolved with treatment. Representative **(c)** and summary **(d)** flow cytometric data demonstrating CXCR5 down‐regulation on blood CD4^+^ T cells in active tattoo uveitis only. Longitudinal samples of Patient 1 and Patient 2 are shown by an open circle and open triangle respectively. **(e)** Frequency of ICOS^+^ CD45RA^−^ CD4^+^ T cells and **(f)** CXCR5^+^ ICOS^+^ CD4^+^ T cells in active and inactive tattoo uveitis, sarcoid uveitis, extra‐ocular sarcoidosis, tattoo sarcoidosis with uveitis and healthy donors. Representative **(g)** and summary **(h)** flow cytometric data of CXCR5 down‐regulation on blood CD19^+^ B cells in active tattoo uveitis only. The samples were normalised to a single healthy control sample used in each experiment to ensure consistency and comparability of MFIs across multiple experimental runs. **(i, j)** Dynamic changes in CXCR5 expression on cTfh cells and CD19^+^ B cells corresponding to clinical evidence of disease activity and remission induced by treatment with TNF inhibitor adalimumab **(i)** or mycophenolate **(j)**. Patient 1 with TNF inhibitor achieved complete ocular and cutaneous remission after 3 months of adalimumab therapy. Patient 2 with mycophenolate treatment achieved a partial remission, though some low‐grade ocular activity persisted. In summary plots, column heights represent the mean, error bars indicate the standard deviation, and each dot represents a single donor; where replicates exist, the mean value is shown. Data were collected from 2 to 3 independent experiments. Statistical analysis was performed by one‐way ANOVA followed by comparisons of all group means. *P*‐values were corrected using Tukey's method for multiple comparisons. ****P < 0.0001, ***P < 0.001, **P < 0.01, *P < 0.05. All other comparisons were not significant.

Tattoo uveitis is a rare disease. We studied six patients with TU (without sarcoidosis), defined as the presence of anterior, intermediate, posterior or pan‐uveitis associated with tattoo inflammation, in the absence of mediastinal adenopathy or radiological evidence of parenchymal lung disease. Four patients had active disease at the time of sampling, defined according to the Standardisation of Uveitis Nomenclature (SUN) criteria,^14^ two of whom also underwent longitudinal sampling at a timepoint of disease quiescence. The remaining two patients had inactive disease at the time of sampling (Supplementary table 1). Notably, all tattoo inflammation reported in this cohort was restricted to areas of black pigment ink (Figure 1a). In all cases, concurrent tattoo and ocular inflammation were observed on at least one occasion. Manifestations of ocular inflammation included clinical features associated with granulomatous disease (vitreous snowballs and keratic precipitates) as well as non‐specific inflammatory changes such as cystoid macular oedema and retinal vasculitis (Figure 1b).

We analysed PBMCs from patients with active TU and compared them to PBMCs from patients with inactive TU, active sarcoid uveitis (including a subset with tattoo inflammation and lymphadenopathy), active extra‐ocular sarcoidosis, inactive sarcoidosis and healthy controls. Our findings revealed no significant differences in absolute monocyte or lymphocyte counts between the groups (Supplementary figure 1a and b). We observed a reduction in memory B cells in patients with active tattoo uveitis compared to inactive controls, whereas no other significant differences were observed in the proportions of lymphocyte subsets (Supplementary figure 1c–f).

Remarkably, CXCR5^+^ CD45RA^−^ CD4^+^ T cells (circulating follicular helper T cells, cTfh) were almost undetectable in patients with active TU and significantly reduced compared with other groups (Figure 1c and d). This phenotype could indicate either down‐regulation of CXCR5 or loss of this population from the circulation. We identified ICOS^+^ CD4^+^ T cells (Figure 1e), which include cTfh cells.^15^, ^16^ Active TU was associated with fewer CXCR5^+^ ICOS^+^ CD4^+^ T cells (Figure 1f), suggesting down‐regulation. We also considered the possibility that CXCR5^low^ CD4^+^ T cells might be peripheral T helper cells (Tph), a population postulated to be tissue‐resident counterparts of Tfh cells that has been reported in some patients with rheumatoid arthritis and systemic lupus erythematosus.^17^, ^18^, ^19^ Tph resemble cTfh cells with upregulation of IL‐21, CXCL13, ICOS, MAF and PD‐1^hi^ but are CXCR5^lo^. There was no expansion of CXCR5^−^ PD‐1^+^ CD4^+^ T cells in active sarcoidosis compared with healthy controls (Supplementary figure 1g and h).

We also observed down‐regulation of CXCR5 on CD19^+^ B cells in patients with active TU, while there was no such reduction in inactive TU, sarcoidosis or healthy controls (Figure 1g and h). Thus, CXCR5 down‐regulation is specific to active TU, where cTfh cells also become undetectable. Notably, the changes in CXCR5 expression were dynamic and occurred in parallel with TU disease activity. During uveitis flares, marked by retinal vasculitis and tattoo inflammation, CXCR5 down‐regulation on B and cTfh cells was prominent. CXCR5 expression returned to normal during disease remission with TNF inhibitor adalimumab (Figure 1i) or mycophenolate (Figure 1j). Thus, transient CXCR5 down‐regulation on B cells and loss of detectable CXCR5^+^ CD4^+^ T cells in the circulation may serve as a specific marker of disease activity in TU.

### Lymphocyte transcriptome analysis

**Figure 2:**
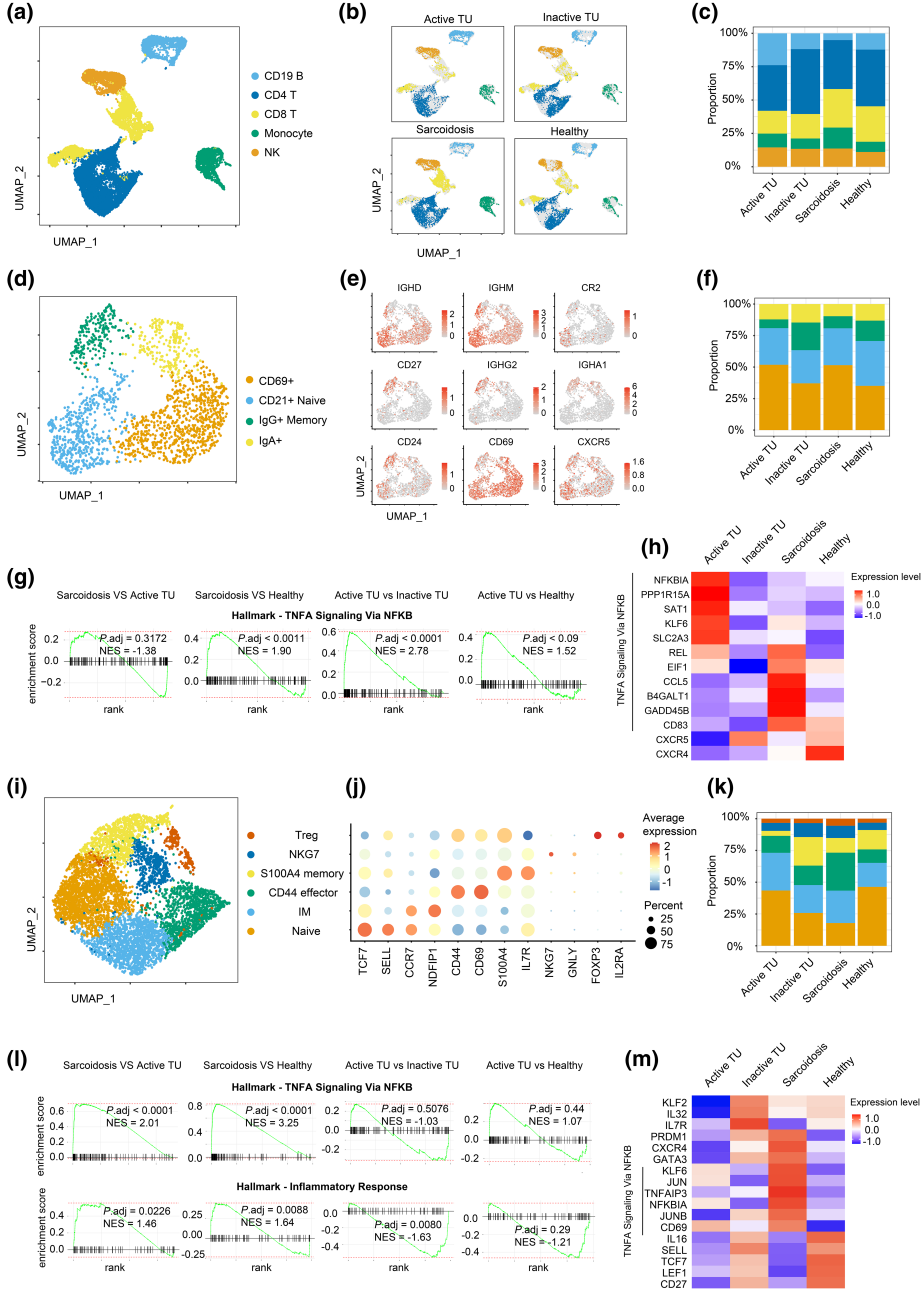
Blood lymphocytes transcriptome analysis for tattoo uveitis and sarcoidosis. **(a, b)** UMAP plots of scRNA‐seq data (merged **a** and split **b**) showing CD4^+^ and CD8^+^ T cells, CD19^+^ B cells, monocytes and NK cells from PBMCs of active and inactive tattoo uveitis, sarcoidosis and healthy donors. **(c)** Frequency of lymphocyte cell subsets from donors with active and inactive tattoo uveitis, sarcoidosis and healthy donors. **(d)** UMAP plot displaying showing CD69^+^, CD21^+^ naive, IgG^+^ memory and IgA^+^ subsets for B cells. **(e)** UMAP plot displaying relative intensity of expression of select genes *IGHD*, *IGHM*, *CR2*, *CD27*, *IGHG2*, *IGHA1*, *CD24*, *CD69* and *CXCR5* for CD19^+^ B cells. **(f)** Frequency of CD69^+^, CD21^+^ naive, IgG^+^ memory and IgA^+^ B cells in patients with active and inactive tattoo uveitis, sarcoidosis and healthy donors. **(g)** Gene set enrichment analysis plots for ‘Hallmark‐TNFA Signaling via NF‐κB’ in B cells transcriptome between the indicated comparisons with adjusted *P*‐values. **(h)** Heatmap showing the selected transcripts of differently expressed genes for total B cells in donors related to ‘Hallmark‐TNFA Signaling via NF‐κB’ gene set. **(i)** UMAP plot showing Treg, NKG7, S100A4 memory, CD44^+^ effector, intermediate memory (IM) and naive subsets for CD4^+^ T cells. **(j)** Dot plot showing the typical markers of differentially expressed genes for indicated CD4^+^ T‐cell subsets. **(k)** Frequency of indicated blood CD4^+^ T‐cell subsets in patients with active and inactive tattoo uveitis, sarcoidosis and healthy donors. **(l)** Gene set enrichment analysis plots for ‘Hallmark‐TNFA Signaling via NF‐κB’ and ‘Hallmark‐Inflammatory Response’ in CD4^+^ T cells transcriptome between the indicated comparisons with adjusted *P*‐values. **(m)** Heatmap showing the differently expressed genes for total CD4^+^ T cells in different donors.

Next, we performed single‐cell RNA‐sequencing analysis on PBMCs isolated from active and inactive TU patients, sarcoidosis patients and age‐matched healthy donors. Major cell subsets were identified using established markers: CD4^+^ T cells (*CD3* ^+^ *CD4* ^+^), CD8^+^ T cells (*CD3* ^+^ *CD8* ^+^), B cells (*CD19* ^+^ *CD79* ^+^), NK cells (*CD3* ^−^ *NKG7* ^+^) and monocytes (*SPI1* ^+^ *CD14* ^+^ or *FCGR3A* ^+^) (Figure 2a, Supplementary figure 2a). Cell subset percentages were comparable between active and inactive TU (Figure 2b and c).

Naive and memory B cell subsets were present in similar proportions across all patient groups (Figure 2d–f, Supplementary figure 2b), with a small relative reduction in IgG^+^ memory B cells in active TU and sarcoidosis compared with healthy donors. Gene set enrichment analysis (GSEA) revealed significant enrichment of genes related to ‘TNF alpha signalling via NF‐κB’ in patients with active TU and sarcoidosis compared with controls (Figure 2g). Consistent with our protein expression data, scRNAseq showed reduced *CXCR5* expression in B cells from active TU patients compared to those in remission (Figure 2h).

CD4^+^ T cell subsets were also similarly represented in patients with active and inactive TU, although we observed a small increase in CD44^+^ effector cells in the sarcoidosis group compared with healthy donors (Figure 2i and k, Supplementary figure 2c). GSEA identified enriched ‘TNF alpha signalling via NF‐κB’ and ‘Inflammatory response’ signatures in CD4^+^ T cells from sarcoidosis, but not active TU patients, compared to healthy donors (Figure 2l). Additionally, CD4^+^ T cells from sarcoidosis patients exhibited increased *PRDM1* and *GATA3* expression, while naive markers *SELL*, *TCF7* and *LEF1* were reduced in sarcoidosis and active TU compared with healthy donors (Figure 2m). Key markers of Tph cells,^17^, ^18^, ^19^ including *TOX*, *MAF*, *TIGIT*, *IFNG*, *BATF* and *CD40LG*, were expressed at low levels (Supplementary figure 2d).

### Clonally expanded cytotoxic CD8^+^ T cells only in sarcoidosis

**Figure 3:**
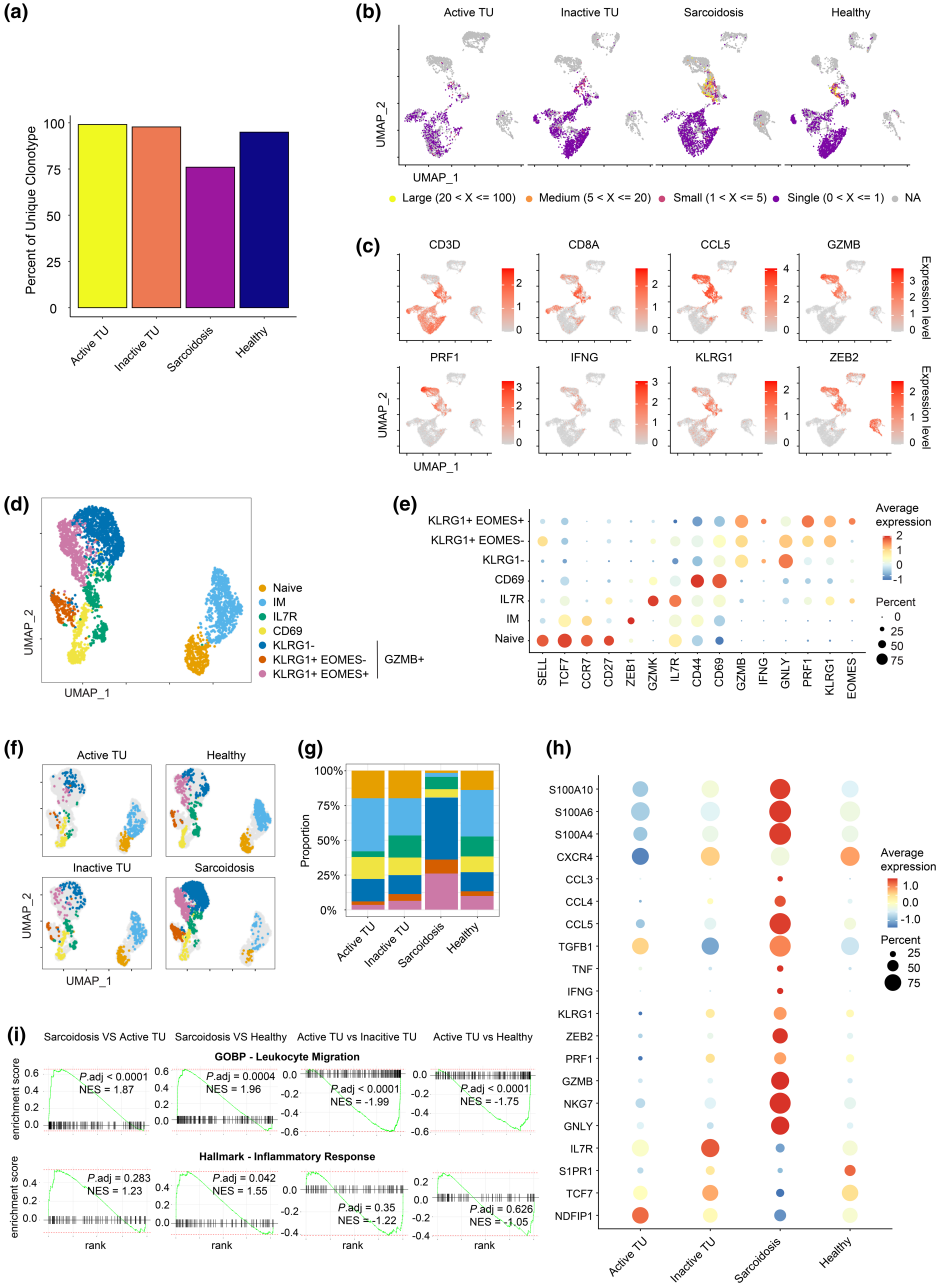
Clonally expanded cytotoxic CD8^+^ T cells in sarcoidosis. **(a)** Percentages of unique T cell clonotypes across four different samples. **(b)** Representative UMAP plots showing the different T cell clonotypes according to the sizes (large—20 < X ≤ 100; medium—5 < X ≤ 20; small—1 < X ≤ 5; single—0 < X ≤ 1). Same clonotypes are defined by cells sharing the same VDJC gene segments and CDR3 regions for both *TCRA* and *TCRB* chains. **(c)** Relative expression intensity of *CD3D*, *CD8A*, *CCL5*, *GZMB*, *PRF1*, *IFNG*, *KLRG1* and *ZEB2* genes. **(d)** Merged UMAP plot displaying naive, CM, IL7R^+^, CD69^+^, KLRG1^−^, KLRG1^−^ EOMES^−^ and KLRG1^+^ EOMES^+^ CD8^+^ T‐cell subsets. **(e)** Relative expression of selected differentially expressed genes for each CD8^+^ T‐cell subsets. **(f, g)** Split UMAP plots **(f)** and frequencies **(g)** of indicated CD8^+^ T‐cell subsets from patients with active and inactive tattoo uveitis, sarcoidosis and healthy donors. **(h)** Dot plot showing the selected transcripts of differently expressed genes for total CD8^+^ T cells in different donors. **(i)** Gene set enrichment analysis plots for ‘GOBP ‐ Leukocyte Migration’ and ‘Hallmark‐Inflammatory Response’ in CD8^+^ T cells transcriptome between the indicated comparisons with adjusted *P*‐values.

One outstanding question in sterile granulomatous diseases, including sarcoidosis and TU, is whether they are driven by antigen‐specific T‐cell responses. Previously, T cells accumulating in the lungs of sarcoidosis patients were reported to exhibit restricted TCR variable gene segment usage.^20^, ^21^, ^22^ We examined TCR usage by T cells from each group using single‐cell sequencing. In both active and inactive TU, most T‐cell clonotypes were unique, with minimal evidence of clonal expansion (Figure 3a). The failure to detect clonal expansion could reflect the relatively small number of cells sampled. Notably, some T‐cell clonotypes are shared between active and inactive TU patients but not with healthy controls, raising the possibility of a common antigenic trigger (Supplementary figure 2e, Supplementary table 2). By contrast, T cells from sarcoidosis patients exhibited clonal expansion of terminally differentiated CD8^+^ effectors that expressed high levels of effector genes, including *CCL5*, *GZMB*, *PRF1*, *IFNG*, *KLRG1* and *ZEB2* (Figure 3b and c).

Within the CD8^+^ T‐cell population, we identified seven distinct subclusters, including naive (*SELL* ^+^ *CCR7* ^+^), IM (intermediate memory; *SELL* ^−^ *CCR7* ^+^),^23^ IL7R^+^ memory (*IL7R* ^+^ *GZMK* ^+^) and CD69^+^ effector (*CD69* ^+^ *CD44* ^+^) CD8^+^ T cells. Additionally, we distinguished three clusters of cytotoxic GZMB^+^ CD8^+^ T cells based on varying levels of *KLRG1* and *EOMES* expression: *KLRG1* ^−^; *KLRG1* ^+^ *EOMES* ^−^; and *KLRG1* ^+^ *EOMES* ^+^ (Figure 3d and e). These *GZMB* ^+^ CD8^+^ T‐cell subsets expressed high levels of cytotoxicity‐related genes, including *ZEB2*, *NKG7*, *GZMB*, *GNLY*, *FGFBP2*, *CTSW* and *EFHD2*, as well as chemokine ligands *CCL3* and *CCL5* (Supplementary figure 2f).

The distribution of CD8^+^ T‐cell subsets was similar between active and inactive TU. Compared to inactive TU, active TU patients had a possible small increase in intermediate memory and CD69^+^ CD8^+^ effectors, which expressed *CXCR4*, *CCR6*, *GATA3*, *TNF* and *RUNX3* as well as *NR4A2*, *NR4A3*, *BTG1*, *ZFP36L1* and *ZFP36L2* upregulated after TCR stimulation.^24^, ^25^, ^26^ Interestingly, compared to healthy donors, active TU was also associated with a reduction in IL7R^+^ CD8^+^ T cells, which also expressed *GZMK* but not *GZMB* (Figure 3f and g, Supplementary figure 2f), a phenotype that has been reported for CD8^+^ T cells recovered from various sites of inflammation.^27^

Remarkably, the evidence for CD8^+^ T‐cell activation was more compelling in sarcoidosis than in active TU. In sarcoidosis patients, CD8^+^ T cells were predominantly *GZMB* ^+^, whereas in active TU and healthy controls, the majority of CD8^+^ T cells consisted of naive and memory T cells (Figure 3f and g). Compared to healthy donors, CD8^+^ T cells in sarcoidosis patients had increased activity, indicated by elevated expression of cytotoxicity‐related genes (*GNLY*, *NKG7*, *GZMB*, *PRF1*, *ZEB2* and *KLRG1*), cytokines (*TNF*, *IFNG* and *TGFB1*), chemokine ligands (*CCL3*, *CCL4* and *CCL5*) and S100 family proteins (*S100A4*, *S100A6*, *S100A10*) (Figure 3h). GSEA revealed that CD8^+^ T cells from sarcoidosis patients had increased expression of genes related to ‘Leukocyte Migration’ compared to healthy donors, while CD8^+^ T cells from active TU exhibited the opposite pattern (Figure 3i). Furthermore, CD8^+^ T cells in sarcoidosis patients exhibited significant enrichment of ‘Inflammatory Response’‐related genes, which was not observed in active TU compared with healthy donors (Figure 3i).

### Enhanced pro‐inflammatory and EMT signatures in monocytes

Monocytes and monocyte‐derived cells are indispensable for granuloma formation.^12^, ^13^ In addition to driving inflammation by activating T cells and secreting pro‐inflammatory cytokines, circulating monocytes migrate into tissue, where they differentiate into macrophages or dendritic cells. These differentiated cells are postulated to serve as a central scaffold for other cell types.^12^, ^28^, ^29^ Furthermore, monocytes in granulomas undergo specific epithelioid transition (also known as mesenchymal‐epithelial transition, MET).^2^ We sought to determine whether signatures of these phenotypes were evident in circulating monocytes.

**Figure 4:**
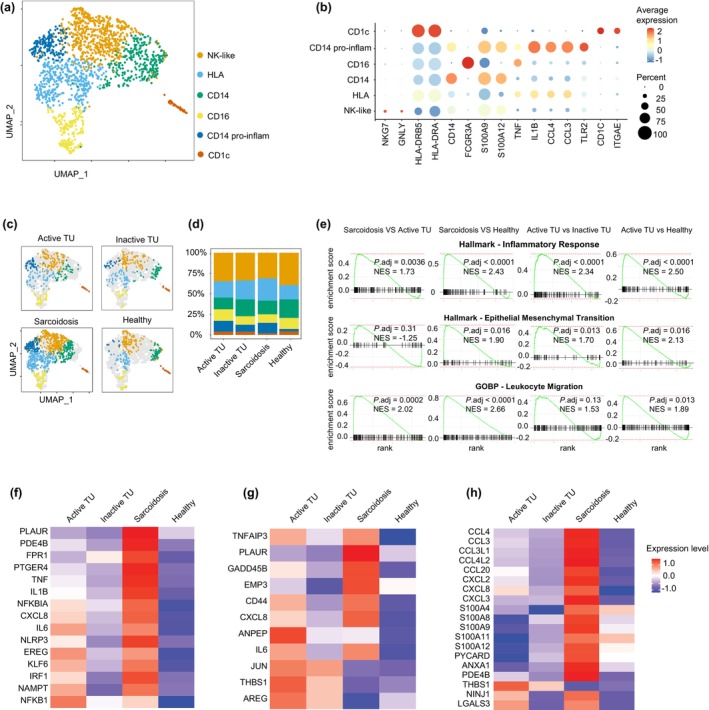
Pro‐inflammatory and EMT signatures in sarcoidosis and active tattoo uveitis monocytes. **(a)** Merged UMAP plot displaying NK‐like, HLA+, IL14^+^, CD16^+^, IL14^+^ pro‐inflammatory and CD1c^+^ subsets for monocytes. **(b)** Relative expression of selected differentially expressed genes for each monocyte subsets. **(c, d)** Split UMAP plots **(c)** and frequencies **(d)** of indicated monocyte subsets from patients with active and inactive tattoo uveitis, sarcoidosis and healthy donors. **(e)** Gene set enrichment analysis plots for the different pathways in monocytes transcriptome between the indicated comparisons with adjusted *P*‐values. **(f–h)** Heatmaps showing the differently expressed genes for the monocytes in different groups than healthy donors related to ‘Hallmark ‐ Inflammatory Response’ **(f)** ‘Hallmark ‐ Epithelial Mesenchymal Transition’ **(g)** and ‘GOBP ‐ Leukocyte Migration’ gene sets **(h).**

We identified six monocyte subclusters: classical CD14^+^ monocytes (*CD14* ^+^ *FCGR3A* ^−^), CD14^+^ pro‐inflammatory monocytes (*CD14* ^+^ *FCGR3A* ^−^ *IL1B* ^+^), CD16^+^ monocytes (*CD14* ^−^ *FCGR3A* ^+^), HLA^+^ monocytes (*HLA‐DRA* ^+^), NK‐like monocytes (*NKG7* ^+^ *GNLY* ^+^) and a tiny population of CD1c^+^ dendritic cells (*CD1C* ^+^ *ITGAE* ^+^) (Figure 4a and b). Classical monocytes exhibit high expression of S100A family members (*S100A8*, *S100A9*, *S100A10*, *S100A11* and *S100A12*) (Supplementary figure 3a). Notably, CD14^+^ pro‐inflammatory monocytes (*CD14* ^+^ *FCGR3A* ^−^ *IL1B* ^+^) are rare in healthy donors but increased in patients with active TU and sarcoidosis compared with healthy donors (Figure 4c and d). This subset expresses many chemokine ligands (*CCL4L2*, *CCL4*, *CCL3*, *CCL3L1*, *CXCL2, CXCL8* and *CCL20*), inflammatory cytokines (*IL6*, *IL1B* and *TNF*), NF‐κB regulators (*NFKB1*, *NFKB2*, *NFKBIZ*, *NFKBID* and *TNFAIP3*) and *TLR2* (Supplementary figure 3a). Consistently, monocytes in sarcoidosis patients and, to a lesser extent in active TU, exhibited significantly increased enrichment of genes related to the ‘Inflammatory Response’, including *IL1B*, *CXCL8* and *TNF* (Figure 4e and f).

While MET is a hallmark of tissue granuloma formation,^1^, ^7^ blood monocytes in both active TU and sarcoidosis exhibited epithelial–mesenchymal transition (EMT) signatures (Figure 4e), including significantly increased expression of *GADD45B*, *CD44*, *CXCL8*, *ANPEP* and *THBS1*,^30^, ^31^, ^32^, ^33^ compared with healthy donors (Figure 4g). *ZEB2* and *SNAIL* gene family members, known to drive EMT,^34^ were selectively expressed in a small subset of monocytes from sarcoidosis patients (Supplementary figure 3c). EMT is characterised by loss of cell–cell adhesion and acquisition of migratory capacity. TNF, an inducer of EMT, is abundantly expressed by monocytes from both sarcoidosis and TU patients (Figure 4f, Supplementary figure 3b), which might help explain the therapeutic efficacy of TNF inhibition in these disorders.^20^

Finally, consistent with the adoption of EMT signatures by monocytes, we noted changes related to migration and chemotaxis (Figure 4e). Specifically, monocytes from patients with sarcoidosis showed significant upregulation of chemokines, including *CCL3*, *CCL4*, *CCL20*, *CXCL8* and *CXCL2*, which were not observed in active TU (Figure 4h, Supplementary figure 3d).

## Discussion

Until now, TU has been considered a rare subset of sarcoidosis.^5^ While we have identified some transcriptional characteristics shared by TU and sarcoidosis, there are important differences, and our findings therefore provide important insights into this rare condition that have implications for resolving the heterogeneity of sterile granulomatous disease. The most obvious and surprising distinction was absent CXCR5^+^ CD4^+^ T cells. We cannot be certain whether this represents CXCR5 down‐regulation or loss of cTfh cells from the circulation. Down‐regulation of CXCR5 was also observed on B cells in patients with active TU but not in those with quiescent disease or in sarcoidosis, supporting the former explanation.

CXCR5 is normally expressed on B cells and the Tfh subset of CD4^+^ T cells.^35^, ^36^, ^37^ The interaction of CXCR5 with its cognate chemokine CXCL13 is necessary for B cells and Tfh cells to enter lymph nodes, specifically lymph node follicles.^38^, ^39^ In a lymphadenopathy‐prone murine model (B6/lpr), CXCR5 deletion is associated with reduced lymphadenopathy and splenomegaly.^40^ Thus, CXCR5 down‐regulation in active TU may explain the absence of distant lymphadenopathy, which is also unique among the granulomatous diseases.

Our observations on B cells suggest the inflammatory milieu of TU is associated with CXCR5^+^ down‐regulation. CXCR5^−^ Tfh‐like cells have been observed in several end‐organ sites of inflammatory pathology, including rheumatoid synovitis, lupus nephritis and sarcoidosis pneumonitis. CXCR5^−^ PD‐1^hi^ cells retain transcriptional signatures reminiscent of Tfh cells^17^, ^18^, ^19^ but PD‐1^hi^ CXCR5^−^ CD4^+^ T cells were not expanded in active sarcoidosis, and very few PBMCs from active TU patients expressed a Tph transcriptional signature (*TOX, MAF, TIGIT, CD40LG*). Taken together, these findings suggest that in active TU, there is a complete loss of CXCR5 expression on CD4^+^ T cells, which may lead to egress of CD4^+^ T cells not to lymph nodes, but to non‐lymphoid parenchyma.

The mechanisms that account for the organ involvement in sterile granulomatous diseases (e.g. eye and skin in TU, lung in sarcoidosis, gut in Crohn's disease) remain unclear although the transcriptional signatures might provide some clues. We observed selective upregulation of a small number of monocyte genes implicated in cell migration, including *ANPEP* (CD13) and *THBS1* (thrombospondin‐1). CD13 is a membrane metalloprotease that has been implicated in cell migration via homotypic interactions with endothelium, and via modification via proteolysis of chemokines.^41^, ^42^ *THBS1* in the monocytes can also facilitate cancer metastasis and positively correlates with the mesenchymal characteristics.^33^, ^43^ *THBS1* is expressed by ocular cells and is required for wound healing and immunoregulation.^44^ Whether this phenotype contributes to the unique and restricted pattern of end‐organ pathology that defines TU merits further investigation. Another possibility is that unique microvasculature makes the eye susceptible to recruitment of monocytes from granulomas. This is plausible, since uveitis appears to be a complication of granulomatous disease, irrespective of the cause.^6^, ^45^, ^46^, ^47^, ^48^

Another possibility is that end‐organ disease results from parenchyma‐specific antigenic targets. Indeed, whether lymphocyte responses in sterile granulomas are antigen‐driven at all has been a contentious topic. It is therefore significant that our single cell analysis provides unequivocal evidence for clonal expansion of CD8^+^ T cells in sarcoidosis. Previous studies have pointed to possible oligoclonal responses in end‐organs of sarcoidosis patients, including CD4^+^ cells in lung and CD8^+^ T cells in cerebrospinal fluid.^20^, ^21^, ^49^ Contrary to assumptions that TU is somehow antigen‐driven by tattoo pigment, we found much weaker evidence of clonal expansion in this group despite clear evidence for T‐ and B‐cell activation.

Finally, in addition to lymphocyte analysis, we also observed that both active TU and sarcoidosis are characterised by an EMT transcriptional signature in monocytes. This is a striking finding, since the opposite transition occurs consistently in tissue monocytes and is one of the defining characteristics of granulomas. EMT occurs concurrently with the adoption of an inflammatory phenotype. Considering the findings for transcriptional changes in granulomas from patients with sarcoidosis or TB points to the possibility that granulomas serve as a depot of lymphocytes and monocytes.^10^, ^11^ When monocytes become activated, the granuloma becomes less organised via EMT and changes in chemokine ligand expression. Lymphocytes and monocytes are then able to migrate to distant sites. TU is distinguished from sarcoidosis because failure to upregulate CXCR5 means that migration of liberated cells does not involve lymph nodes. A strength of our study is the comparison of patients with active and inactive disease, including longitudinal analysis of patients during phases of disease activity and quiescence. A major limitation of our study, however, was the small sample size, a consequence of the rarity of TU. Further efforts will be required to replicate our findings at other centres.

## Methods

### Patient and public involvement

Patients with sarcoidosis were recruited from the Sarcoidosis Quaternary Referral Clinic (ACT Health, Canberra, Australia) according to the following criteria: (1) Age over 18 years and (2) A diagnosis of sarcoidosis in accordance with criteria outlined in the Joint Statement of the American Thoracic Society (ATS), the European Respiratory Society (ERS) and the World Association of Sarcoidosis and Other Granulomatous Disorders (WASOG).^50^ Patients were excluded if their granulomatous disease could be attributed to another aetiology such as Crohn's disease or Blau syndrome. Infectious causes of granulomatous inflammation were excluded on tissue or fluid culture and/or interferon‐gamma release assays for *Mycobacterium tuberculosis*. The organs affected in the sarcoidosis group consisted of pulmonary disease/thoracic lymphadenopathy (*n* = 50), skin granulomas (*n* = 12), uveitis (*n* = 18), liver (*n* = 7), spleen (*n* = 11), nervous system (*n* = 6), heart (*n* = 7), salivary gland (*n* = 9), bone and joint (*n* = 9) and extra‐thoracic lymphadenopathy (*n* = 15). The average age at the time of diagnosis was 48.2 years (SD ± 12.1). Females made up 58.9% of the group.

Tattoo uveitis (without sarcoidosis) was defined as the presence of anterior, intermediate, posterior or pan‐uveitis associated with cutaneous lesions suggestive of tattoo inflammation, in the absence of mediastinal adenopathy or pulmonary parenchymal changes. These patients were recruited from tertiary uveitis centres (ACT Health, Royal Victorian Eye and Ear Hospital) and from a private uveitis clinic (Melbourne, Victoria). All patients underwent a complete uveitis and sarcoidosis evaluation with laboratory and radiological investigations to exclude other differential diagnoses for the pattern of uveitis and to exclude systemic sarcoidosis. Disease activity at the time of clinical assessment was determined by the treating clinician; the threshold for ‘active’ disease was defined according to the Standardisation of Uveitis Nomenclature (SUN) criteria.^14^ The average age of active tattoo uveitis patients was 31.5 years (SD ± 6.4), and 33.3% of them were female.

Two patients had both uveitis and tattoo inflammation, as well as histological and radiological evidence of sarcoidosis in their thoracic lymph nodes. These two patients were included in the sarcoidosis group. Healthy controls included 30 subjects with a mean age of 49.5 years (SD ± 12.7), of whom 63.3% were female. The study was approved by the ACT Health Human Research Ethics Committee (ETH.1.15.015). Written informed consent was obtained from each patient at the time of enrolment. All clinical photography was obtained with patient consent for publication.

### Flow cytometric immunophenotyping of circulating lymphocytes

Frozen cells were thawed, stained and analysed by flow cytometry. Samples were batched to ensure that all samples from any individual patient were assayed together. For analysis of T helper cells, samples were labelled using anti‐CD4 APC‐Cy7 (clone RPA‐T4), anti‐CD45RA Pacific Blue (HI100), anti‐CXCR5 PerCP‐Cy5.5 (J252D4), anti‐CXCR3 BV510 (G025H7) (Biolegend, San Diego, USA), anti‐CD57 FITC (HNK‐1), anti‐CCR6 PE (11A9) (BD Biosciences, San Diego, CA, USA), anti‐PD‐1 Biotin (J105) and Streptavidin APC (Thermo Fisher Scientific, Waltham, USA). For Treg cells, surface staining was performed using anti‐CD4 APC‐Cy7, anti‐CD45RA Pacific Blue, anti‐CD127 FITC (A019D5) (Thermo Fisher Scientific) and anti‐CD25 APC (2A3) (BD Biosciences). For B cell analysis, cells were first blocked with FcR Blocking Reagent (Biolegend) and then stained with anti‐IgM FITC (Dako, Santa Clara, USA), anti‐IgD PE (IA6‐2), anti‐CD24 BV510 (ML5), anti‐CD38 V450 (HB7), anti‐CD27 APC (L128), anti‐CD19 PeCy7 (SJ25C1) (BD Biosciences), anti‐CD21 PerCP‐Cy5.5 (Bu32) and anti‐CD10 APC‐Cy7 (H110a) (Biolegend). For further analysis of CXCR5 expression on different cell populations, cells were labelled with anti‐CXCR5 AF647 (RF8B2), anti‐CD19 BV510 (SJ25C1), anti‐CCR7 PeCy7 (3D12), anti‐CD45 RA PerCP‐Cy5.5 (HI100), anti‐PD‐1 BV421 (EH12.1), anti‐CD57 FITC (HNK‐1) (BD Biosciences), anti‐CD4 APC‐Cy7 (Biolegend), anti‐ICOS Biotin (ISA3) and Streptavidin APC (Thermo Fisher Scientific). For all surface staining, cells were incubated for 20 min at 4°C; for intracellular staining, cells were incubated for 30 min at 4°C.

Stained cells were washed twice (5 min, 350 g) with sterile phosphate buffered saline (PBS) containing 2% heat‐inactivated FBS and fixed (Cytofix, BD Biosciences). Samples were resuspended in the same buffer (PBS with 2% inactivated FBS) for acquisition. All samples were acquired using a FACSCanto II (BD Biosciences). Analysis of FACS data was performed using the FlowJo software (Version 10.1).

### Single‐cell RNA‐sequencing

Sorted live PBMCs were prepared from two patients with active tattoo uveitis, three patients with convalescent tattoo uveitis, three patients with active sarcoidosis and three age‐matched healthy donors. Every sample from each group was labelled with different TotalSeq‐C hashtag antibodies for pooling (Biolegend). Single‐cell gene expression and TCR VDJ libraries were prepared by Chromium Next GEM Single Cell 5’ Kit v2 Dual Index and Single Cell Human TCR Amplification Kit (10× Genomics, Pleasanton, CA, USA) with the help of the Biomolecular Resource Facility at ANU. Libraries were then sequenced on a NovaSeq 6000 sequencer (Illumina, San Diego, CA, USA) with 2 × 50 bp paired‐end reads. 10× Cell Ranger (10× Genomics, v6.0.1) was utilised to align the reads to the human genome (GRCh38‐2020‐A) using default parameters.

### Sequencing data analysis

The filtered feature‐barcode matrices were imported into R for downstream analysis using the Seurat package (v4.1.0).^51^ Doublets and dead cells were filtered out by quality control. Cells with the number of detected genes between 200 and 3500 and the proportion of mitochondrion gene reads < 10% were selected for further analysis. All gene expression count data were carried out with SCTransform (V2, regulation) for normalisation and variance stabilisation. Antibody hashtag data were normalised by centred log‐ratio (CLR) transformation, and cells with double and negative labels were removed.

A total of 3085 cells from two active tattoo uveitis, 3142 cells from three convalescent tattoo uveitis, 5153 cells from three sarcoidosis and 3804 cells from three healthy donors were submitted for the integration analysis to compare the samples between different groups. Features that are repeatedly variable across datasets were selected as anchors, while ribosomal genes and immunoglobulin and TCR variable genes are ignored to avoid unwanted effects.^52^ After the integration, dimensionality reduction was performed with 20 principal components (PCs), followed by cell cluster identification with a resolution of 0.6, and differentially expressed genes for every cell cluster were identified.

We further partitioned the monocytes, CD4^+^ and CD8^+^ T cells, B cells and NK cells separately as described,^53^; dimensionality reduction and cluster identification were performed, followed by the calculation of markers (differentially expressed genes) using the parameters (min.pct > 0.1, log (FC) > 0.2, *SCT* assay, *P*.val.adj < 0.05 and the default wilcoxon rank sum test), and differentially expressed genes were selected for the heatmap analysis.

Gene set enrichment analysis was performed by fgsea package (V.1.23.4). Genes expressed by more than 5% of cells in either of the two groups were ranked by log fold‐change of the average expression. Gene set enrichment analysis tests were performed based on the Molecular Signatures Database (MSigDB) with the threshold of adjusted *P*‐value < 0.05.

Filtered annotations assembled by the Cell Ranger were used for TCR clonotype analysis by the ScRepertoire package (v1.3.5).^53^ Cells with the same VDJC gene segments and the CDR3 region for *TCRA* and *TCRB* chains were viewed as the same clonotype. The unique clonotype proportions between different samples were calculated, and the same clonotypes between two groups were analysed by the scatterClonotype function.

Single‐cell RNA‐seq data with gene expression and VDJ profiling data were deposited in GEO under GSE232802.

### Statistical analysis

Statistical tests were performed using Prism (GraphPad V9.3.1). As indicated in the figure captions, two‐tailed unpaired *t*‐tests or one‐way ANOVA with Tukey's multiple comparison tests were performed according to the experimental designs. *P < 0.05; **P < 0.01; ***P < 0.001; ****P < 0.0001.

## Conflict of interest

The authors have no competing financial interests to declare.

## Author contributions

**Yuwei Hao:** Conceptualization; data curation; formal analysis; investigation; methodology; software; validation; visualization; writing – original draft; writing – review and editing. **Anthea Anantharajah:** Conceptualization; data curation; formal analysis; funding acquisition; investigation; project administration; resources; validation; visualization; writing – original draft; writing – review and editing. **Jane M Wells:** Resources; visualization; writing – original draft. **Lyndell L Lim:** Resources; writing – original draft. **Anthony JH Hall:** Resources; writing – original draft. **Gary YJ Chew:** Resources; writing – original draft. **Matthew C Cook:** Conceptualization; data curation; funding acquisition; project administration; resources; supervision; writing – original draft; writing – review and editing.

## Ethics approval

This study and associated sub‐studies were approved by the ACT Health and Australian National University Human Research Ethics Committees.

## Patient consent for publication

Consent was obtained directly from patients.

## Patient and public involvement

Sarcoidosis patients were involved in the instigation and funding of this research.

## Supporting information


Supporting information

## Data Availability

Data are available from the corresponding author upon reasonable request.
