# Supplementary material for: Loss of CXCR5 expression and monocyte epithelial–mesenchymal transition are blood‐borne signatures of sterile granulomatous diseases
Source: Clin Transl Immunology. 2025 Jun 3;14(6):e70039. doi: 10.1002/cti2.70039 (PMC12133384; doi:10.1002/cti2.70039)
Supplement: Supplementary file 1 — Supporting information [file CTI2-14-e70039-s001.pdf]

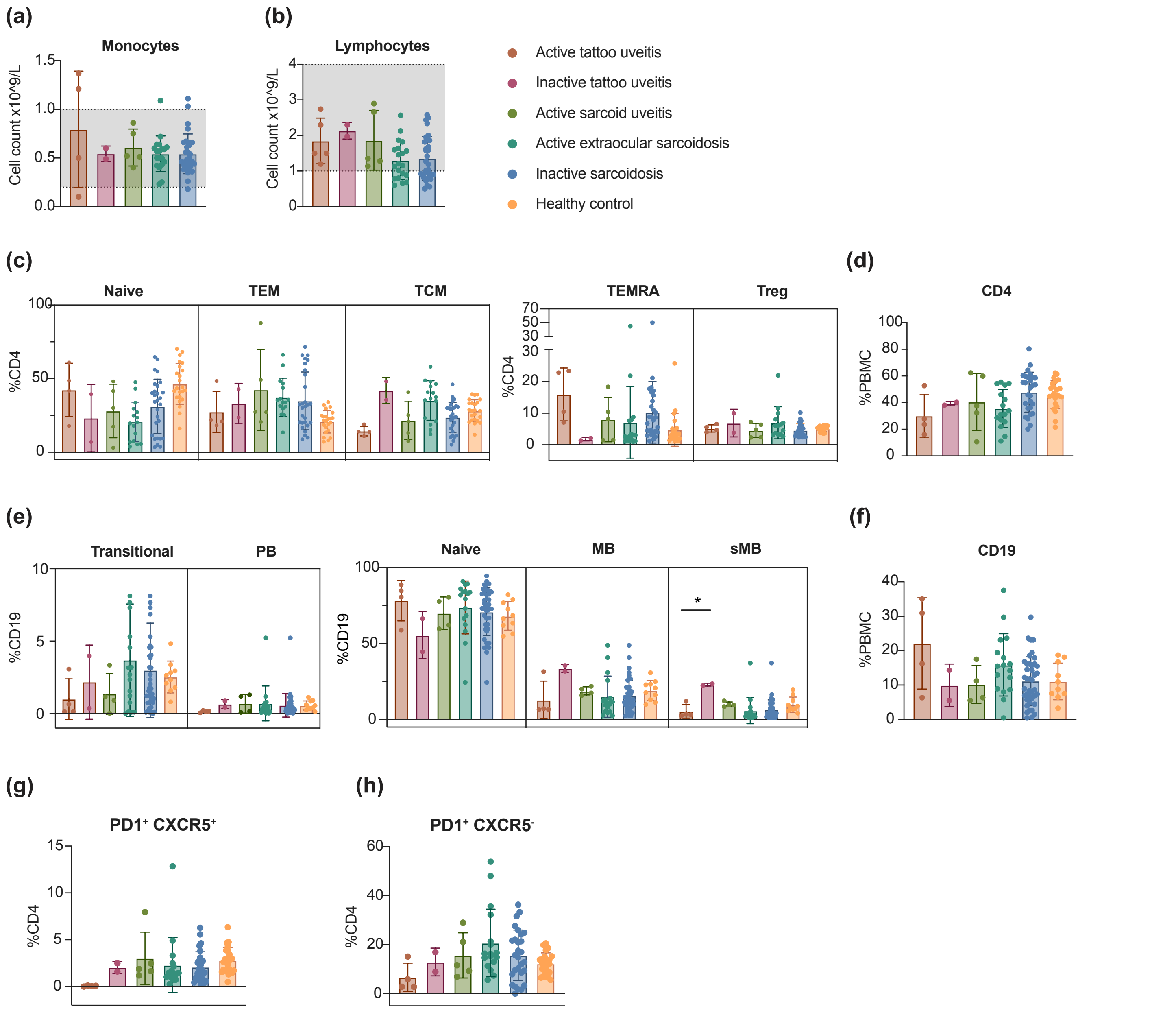

Supplementary figure 1. Flow cytometric analyses of lymphocytes in patients with granulomatous diseases. **(a-b)** Summary of flow cytometry, showing blood monocyte **(a)** and lymphocyte **(b)** counts in patients with active and inactive tattoo uveitis, active sarcoid uveitis, active extraocular sarcoidosis and healthy controls, shaded areas indicate the reference range from a diagnostic assay **(c)** Percentages of naive ($CD45RA^+$ $CCR7^+$), effector memory ($CD45RA^-$ $CCR7^-$), central memory ($CD45RA^-$ $CCR7^+$), Temra ($CD45RA^+$ $CCR7^-$) and Treg ($CD25^+$ $CD127^-$) cells in $CD4^+$ T cells for indicated groups. **(d)** Percentages of total $CD4^+$ T cells for indicated groups. **(e)** Percentages of B cell subsets, including transitional (IgM^{hi} $CD38^{hi}$) and plasmablasts (PB, IgM^- $CD38^{hi}$), naive ($CD27^-$), total memory (MB, $CD19^+$ $CD27^+$) and switched memory (sMB, IgD^- IgM^- $CD27^+$), across indicated groups. **(f)** Percentages of total $CD19^+$ B cells for indicated groups. Percentages of PD-1^+ $CXCR5^+$ **(g)** and PD-1^+ $CXCR5^-$ **(h)** $CD4^+$ T cells for indicated groups. In all summary plots, column heights represent the mean, error bars indicate the standard deviation, and each dot represents a single donor. Data were collected from 2-3 independent experiments. Statistical analysis was performed using one-way ANOVA followed by multiple group comparisons. *P*-values were adjusted using Tukey's method for multiple comparisons. **** $P < 0.0001$, *** $P < 0.001$, ** $P < 0.01$, * $P < 0.05$; all other comparisons were not significant.

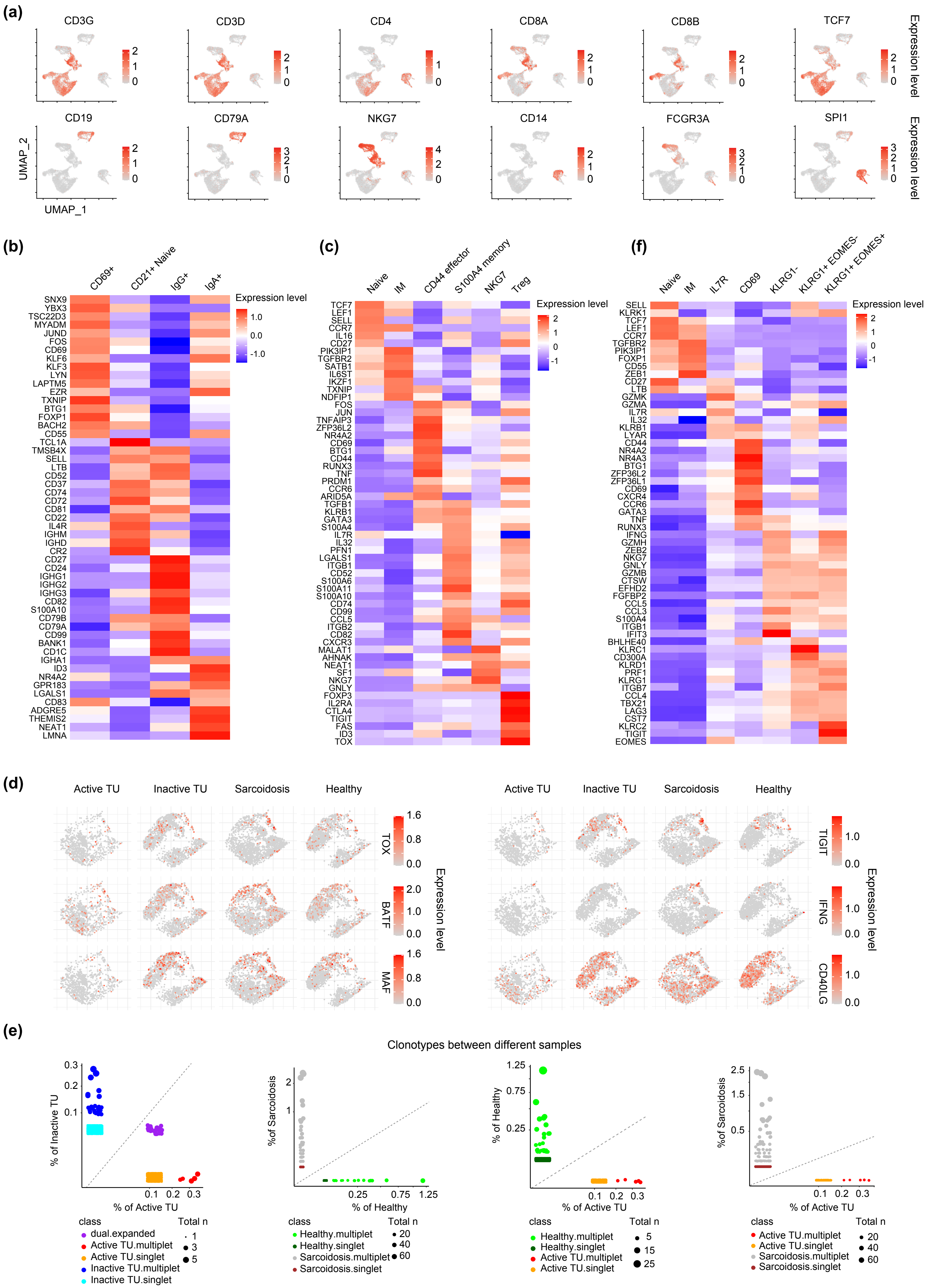
(a)
CD3G
CD3D
CD4
CD8A
CD8B
TCF7
CD19
CD79A
NKG7
CD14
FCGR3A
SPI1
UMAP_2
UMAP_1
Expression level
(b)
CD69+
CD21+ Naive
IgG+
IgA+
Expression level
SNX9 YBX3 TSC22D3 MYADM JUND FOS CD69 KLF6 KLF3 LYN LAPTM5 EZR TXNIP BTG1 FOXP1 BACH2 CD55 TCL1A TMSB4X SELL LTB CD52 CD37 CD74 CD72 CD81 CD22 IL4R IGHM IGHD CR2 CD27 CD24 IGHG1 IGHG2 IGHG3 CD82 S100A10 CD79B CD79A CD99 BANK1 CD1C IGHA1 ID3 NR4A2 GPR183 LGALS1 CD83 ADGRE5 THEMIS2 NEAT1 LMNA
(c)
Naive
IM
CD44 effector
S100A4 memory
NKG7
Treg
Expression level
TCF7 LEF1 SELL CCR7 IL16 CD27 PIK3IP1 TGFBR2 SATB1 IL6ST IKZF1 TXNIP NDFIP1 FOS JUN TNFAIP3 ZFP36L2 NR4A2 CD69 BTG1 CD44 RUNX3 TNF PRDM1 CCR6 ARID5A TGFB1 KLRB1 GATA3 S100A4 IL7R IL32 PFN1 LGALS1 ITGB1 CD52 S100A6 S100A11 S100A10 CD74 CD99 CCL5 ITGB2 CD82 CXCR3 MALAT1 AHNAK NEAT1 SF1 NKG7 GNLY FOXP3 IL2RA CTLA4 TIGIT FAS ID3 TOX
(f)
Naive
IM
IL7R
CD69
KLRG1-
KLRG1+ EOMES-
KLRG1+ EOMES+
Expression level
SELL KLRK1 TCF7 LEF1 CCR7 TGFBR2 PIK3IP1 FOXP1 CD55 ZEB1 CD27 LTB GZMK GZMA IL7R IL32 KLRB1 LYAR CD44 NR4A2 NR4A3 BTG1 ZFP36L2 ZFP36L1 CD69 CXCR4 CCR6 GATA3 TNF RUNX3 IFNG GZMH ZEB2 NKG7 GNLY GZMB CTSW EFHD2 FGFBP2 CCL5 CCL3 S100A4 ITGB1 IFIT3 BHLHE40 KLRC1 CD300A KLRD1 PRF1 KLRG1 ITGB7 CCL4 TBX21 LAG3 CST7 KLRC2 TIGIT EOMES
(d)
Active TU
Inactive TU
Sarcoidosis
Healthy
TOX
BATF
MAF
TIGIT
IFNG
CD40LG
Expression level
(e)
Clonotypes between different samples
% of Inactive TU
% of Active TU
%of Sarcoidosis
% of Healthy
% of Healthy
% of Active TU
%of Sarcoidosis
% of Active TU
class
Total n
dual.expanded
Active TU.multiplet
Active TU.singlet
Inactive TU.multiplet
Inactive TU.singlet
Healthy.multiplet
Healthy.singlet
Sarcoidosis.multiplet
Sarcoidosis.singlet
Active TU.multiplet
Active TU.singlet

Supplementary figure 2. Additional analyses of T and B cell transcripts and clonotypes. **(a)** Plots showing the relative expression levels of the typical markers for $CD4^+$ T cells ($CD3^+$ $CD4^+$), $CD8^+$ T cells ($CD3^+$ $CD8^+$), B cells ($CD19^+$ $CD79^+$), monocytes ($SPI1^+$ $CD14^+$ or $FCGR3A^+$), and NK cells ($CD3^-$ $NKG7^+$). **b-c, f.** Heatmaps of relative expression of selected differentially expressed genes in B cells **(b)**, $CD4^+$ T cells **(c)** and $CD8^+$ T cells **(f)**. **(d)** Feature plots displaying Tph cell gene signatures in $CD4^+$ T cells. **(e)** Scatter plots to compare clonotypes between every two different samples. Dot sizes indicate the cumulative count of clones exhibiting various colours in the expansion patterns. The percentage of clonotypes across all T cell clonotypes is also shown with diagonal lines for equal cell fractions.

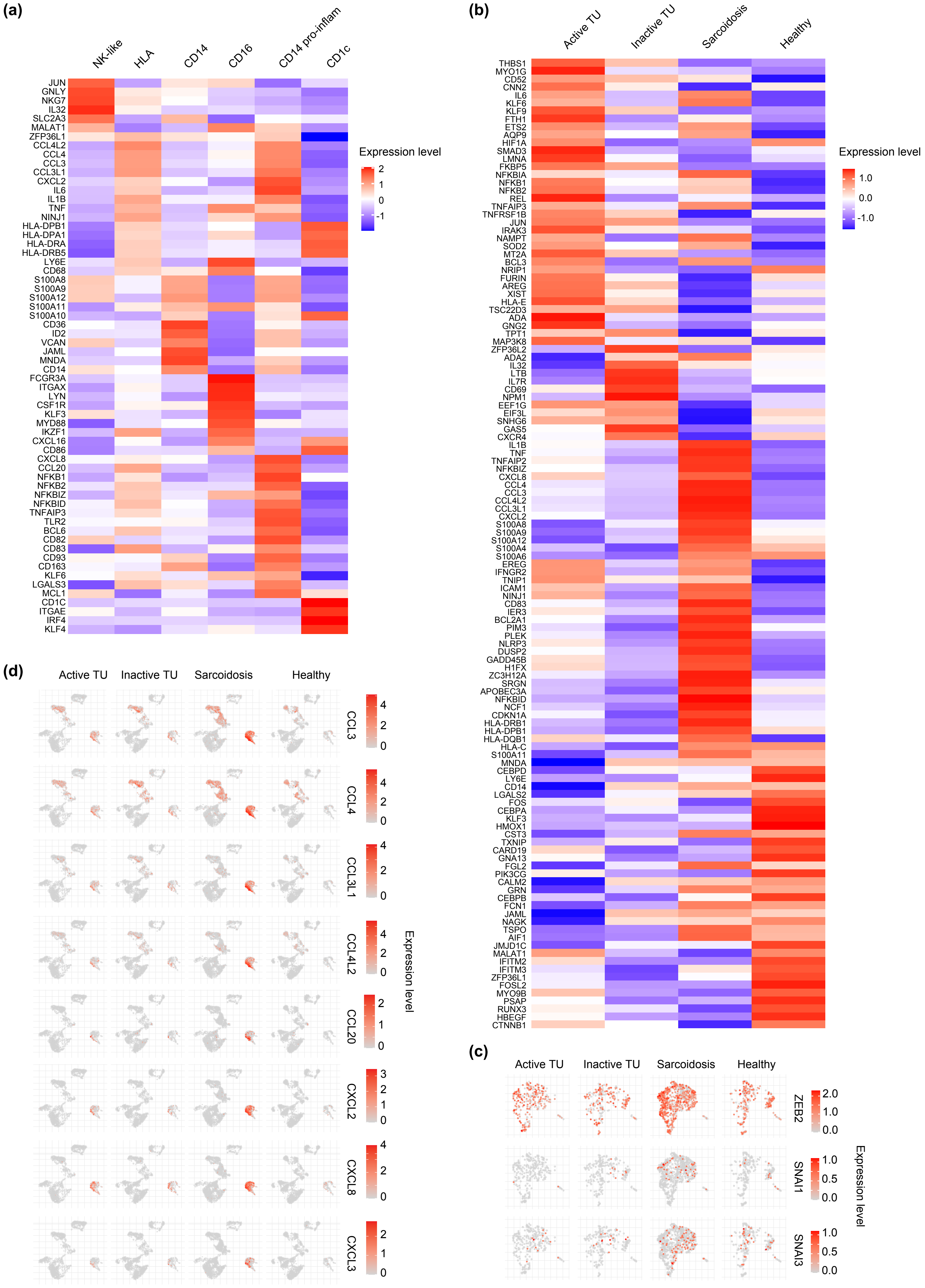

(a)
NK-like
HLA
CD14
CD16
CD14 pro-inflam
CD1c
JUN
GNLY
NKG7
IL32
SLC2A3
MALAT1
ZFP36L1
CCL4L2
CCL4
CCL3
CCL3L1
CXCL2
IL6
IL1B
TNF
NINJ1
HLA-DPB1
HLA-DPA1
HLA-DRA
HLA-DRB5
LY6E
CD68
S100A8
S100A9
S100A12
S100A11
S100A10
CD36
ID2
VCAN
JAML
MNDA
CD14
FCGR3A
ITGAX
LYN
CSF1R
KLF3
MYD88
IKZF1
CXCL16
CD86
CXCL8
CCL20
NFKB1
NFKB2
NFKBIZ
NFKBID
TNFAIP3
TLR2
BCL6
CD82
CD83
CD93
CD163
KLF6
LGALS3
MCL1
CD1C
ITGAE
IRF4
KLF4
Expression level
2
1
0
-1
(b)
Active TU
Inactive TU
Sarcoidosis
Healthy
THBS1
MYO1G
CD52
CNN2
IL6
KLF6
KLF9
FTH1
ETS2
AQP9
HIF1A
SMAD3
LMNA
FKBP5
NFKBIA
NFKB1
NFKB2
REL
TNFAIP3
TNFRSF1B
JUN
IRAK3
NAMPT
SOD2
MT2A
BCL3
NRIP1
FURIN
AREG
XIST
HLA-E
TSC22D3
ADA
GNG2
TPT1
MAP3K8
ZFP36L2
ADA2
IL32
LTB
IL7R
CD69
NPM1
EEF1G
EIF3L
SNHG6
GAS5
CXCR4
IL1B
TNF
TNFAIP2
NFKBIZ
CXCL8
CCL4
CCL3
CCL4L2
CCL3L1
CXCL2
S100A8
S100A9
S100A12
S100A4
S100A6
EREG
IFNGR2
TNIP1
ICAM1
NINJ1
CD83
IER3
BCL2A1
PIM3
PLEK
NLRP3
DUSP2
GADD45B
H1FX
ZC3H12A
SRGN
APOBEC3A
NFKBID
NCF1
CDKN1A
HLA-DRB1
HLA-DPB1
HLA-DQB1
HLA-C
S100A11
MNDA
CEBPD
LY6E
CD14
LGALS2
FOS
CEBPA
KLF3
HMOX1
CST3
TXNIP
CARD19
GNA13
FGL2
PIK3CG
CALM2
GRN
CEBPB
FCN1
JAML
NAGK
TSPO
AIF1
JMJD1C
MALAT1
IFITM2
IFITM3
ZFP36L1
FOSL2
MYO9B
PSAP
RUNX3
HBEGF
CTNNB1
Expression level
1.0
0.0
-1.0
(c)
Active TU
Inactive TU
Sarcoidosis
Healthy
ZEB2
2.0
1.0
0.0
SNAI1
1.0
0.5
0.0
SNAI3
1.0
0.5
0.0
Expression level
(d)
Active TU
Inactive TU
Sarcoidosis
Healthy
CCL3
4
2
0
CCL4
4
2
0
CCL3L1
4
2
0
CCL4L2
4
2
0
CCL20
2
1
0
CXCL2
3
2
1
0
CXCL8
4
2
0
CXCL3
2
1
0
Expression level

Supplementary figure 3. Additional analyses of monocytes transcripts. **(a)** Heatmap shows the relative expression of selected differentially expressed genes in NK-like, HLA^+, $CD14^+$, $CD16^+$, $CD14^+$ proinflammatory and $CD1c^+$ subsets for monocytes. **(b)** Heatmap shows some differently expressed genes for total monocytes from donors with active and inactive tattoo uveitis, sarcoidosis and healthy donors. **(c)** Feature plots showing the expression levels of selected gene signatures of ZEB2, SNAI1 and SNAI3 by monocytes. **(d)** Plots showing the significant upregulations of chemokine ligands by monocytes from total PBMCs in sarcoidosis (*CXCL2*, *CXCL3*, *CXCL8*, *CCL3*, *CCL4*, *CCL20*, *CCL3L1*, *CCL4L2*) and active tattoo uveitis (*CCL3*, *CCL4*, *CCL3L1*, *CCL4L2*) than healthy donor.

Supplementary table 1. Clinical characteristics of TU patients

	Patient 1	Patient 2	Patient 3	Patient 4	Patient 5	Patient 6
Age at sampling	37	25	33	25	27	42
Sex	Male	Male	Female	Male	Female	Male
Ethnicity	Australian European	Australian European	Australian European	Pasifika	Australian European	Australian European
Treatment at sampling	a. Nil	a. Nil	Intraocular corticosteroid	Prednisolone	Nil	Nil
	b. Adalimumab	b. Mycophenolate mofetil		Methotrexate		
Activity at sampling	a. Yes	a. Yes	Yes	Yes	No	No
	b. No	b. +/- †				
Flow cytometry	Y	Y	Y	Y	Y	Y
scRNASeq	N	Y§	Y	Y	Y	Y

† Partial remission was achieved on mycophenolate therapy, although low-grade ocular activity persisted.

§ Only inactive sample for scRNAseq analysis

Supplementary table 2. Shared T cell clonotypes between active and inactive TU patients in the scRNAseq analysis

Clonotypes	No. of cells in active TU	No. of cells in inactive TU
NA_NA_TRBV19.NA.TRBJ1-2.TRBC1_TGTGCCAGTACCCCAACGCAGGTCCGCTATGGCTACACCTTC	1	1
NA_NA_TRBV19.NA.TRBJ2-7.TRBC2_TGTGCCAGTAGTTTCGGGGGTCGATACGAGCAGTACTTC	1	1
NA_NA_TRBV2.NA.TRBJ2-1.TRBC2_TGTGCCAGCACAGTAGATAGCGGGGGAGGGAATGAGCAGTTCTTC	1	1
NA_NA_TRBV20-1.NA.TRBJ2-1.TRBC2_TGCAGTGCTAGCCGGACTAGCGGGGGTACCAATGAGCAGTTCTTC	1	1
NA_NA_TRBV20-1.TRBD2.TRBJ2-1.TRBC2_TGCAGTGCTAGAGAGACACCATGGACTAGCGGGAGCTATAATGAGCAGTTCTTC	1	1
NA_NA_TRBV28.NA.TRBJ2-1.TRBC2_TGTGCCAGCACACCCCCGGCGGGAGTGACGAGCAATGAGCAGTTCTTC	1	1
NA_NA_TRBV28.NA.TRBJ2-2.TRBC2_TGTGCCAGCAGTGTGATCCCGAACACCGGGGAGCTGTTTTTT	1	1
NA_NA_TRBV29-1.NA.TRBJ2-5.TRBC2_TGCAGCGTTGAAGACAGGGAGACCCAGTACTTC	1	1
NA_NA_TRBV29-1.NA.TRBJ2-7.TRBC2_TGCAGCGTTGAAGAGGGCCCAGAGTACGAGCAGTACTTC	1	1
NA_NA_TRBV5-1.NA.TRBJ2-3.TRBC2_TGCGCCAGCAGCCACGGGACGGCTTTAGATACGCAGTATTTT	1	1
NA_NA_TRBV5-5.TRBD1.TRBJ1-4.TRBC1_TGTGCCAGCAGCGAAAGGACAGGGGGCGATGAAAAACTGTTTTTT	1	1
NA_NA_TRBV6-5.TRBD1.TRBJ2-3.TRBC2_TGTGCCAGCAGGGACAGGCGCACAGATACGCAGTATTTT	1	1
NA_NA_TRBV7-2.NA.TRBJ1-1.TRBC1_TGTGCCAGCAGCCTCAACGGGGGGTCAGCTTTCTTT	1	1
NA_NA_TRBV7-2.NA.TRBJ1-5.TRBC1_TGTGCCAGCAGCCCCCGGGAGGGAAATCAGCCCCAGCATTTT	1	1
NA_NA_TRBV7-2.NA.TRBJ2-1.TRBC2_TGTGCCAGCAGCTTAATATTAGGCGAGCAGTTCTTC	1	1
NA_NA_TRBV7-2.NA.TRBJ2-3.TRBC2_TGTGCCAGCAGCCCCCCCCCGGGATTACGCACAGATACGCAGTATTTT	1	1
NA_NA_TRBV7-2.NA.TRBJ2-5.TRBC2_TGTGCCAGCAGCTTAAGTGGCTCGATCCTGGAGACCCAGTACTTC	1	1
NA_NA_TRBV7-2.NA.TRBJ2-5.TRBC2_TGTGCCAGCAGCTTAGGCGGGGGCCAAGAGACCCAGTACTTC	1	1
NA_NA_TRBV7-8.NA.TRBJ2-1.TRBC2_TGTGCCAGCAGCTTTAGGGGAGGAGGAGGGAATGAGCAGTTCTTC	1	1
NA_NA_TRBV7-9.NA.TRBJ2-7.TRBC2_TGTGCCAGCAGCCCCGTAGGGATCAGCTCCTACGAGCAGTACTTC	1	1
NA_NA_TRBV7-9.NA.TRBJ2-7.TRBC2_TGTGCCAGCAGCTTAGCCGGACAGACCTACGAGCAGTACTTC	1	1
TRAV1-1.TRAJ23.TRAC_TGCGCTGTGAACGGTAACCAGGGAGGAAAGCTTATCTTC_TRBV7-2.NA.TRBJ1-5.TRBC1_TGTGCCAGCAGCTTAGGGCAGGTTAGCAATCAGCCCCAGCATTTT	1	1
TRAV20.TRAJ4.TRAC_TGTGCTGTGCAGGCCTTTTCTGGTGGCTACAATAAGCTGATTTTT_TRBV6-2.NA.TRBJ1-1.TRBC1_TGTGCCAGCAGTTATTTGGGAGAAGCTTTCTTT	1	1
TRAV21.TRAJ48.TRAC_TGTGCTGTGAGGGACTTTGGAAATGAGAAATTAACCTTT_TRBV7-8.NA.TRBJ1-2.NA_TGTGCCAGCAGCTTTCGGCAGAGCCTCTATGGCTACACCTTC	1	1
TRAV22.TRAJ20.TRAC_TGTGCTGTTCGTTCTAACGACTACAAGCTCAGCTTT_TRBV10-3.NA.TRBJ2-4.TRBC2_TGTGCCATCAGTGATCAGACACAAAACATTCAGTACTTC	1	1
TRAV27.TRAJ22.TRAC_TGTGCAGGGGCTTCTGGTTCTGCAAGGCAACTGACCTTT_TRBV29-1.NA.TRBJ1-2.TRBC1_TGCAGCGTTGGCCTTAGGGGAGGGAACTATGGCTACACCTTC	1	1
TRAV29/DV5.TRAJ40.TRAC_TGTGCAGCAAGCGACGGATCAGGAACCTACAAATACATCTTT_TRBV14.NA.TRBJ1-1.TRBC1_TGTGCCAGCAGCCGATCCCAGGGGGCCTACACTGAAGCTTTCTTT	1	1
TRAV8-1.TRAJ39.TRAC_TGTGCCGCGGATTATAATGCAGGCAACATGCTCACCTTT_TRBV3-1.TRBD2.TRBJ2-1.TRBC2_TGTGCCAGCAGCCCCGGACTAGCGGGGGGCAGGATTTACAATGAGCAGTTCTTC	1	1
TRAV8-3.TRAJ18.TRAC_TGTGCTGTGGGTCCCTTGAAGAGAGGCTCAACCCTGGGGAGGCTATACTTT_TRBV7-2.NA.TRBJ2-5.TRBC2_TGTGCCAGCAGCTACCGGGAGACCCAGTACTTC	1	1
